# Biodiversity and Spatiotemporal Distribution of Spontaneous Vegetation in Tangdao Bay National Wetland Park, Qingdao City, China

**DOI:** 10.3390/ijerph191811665

**Published:** 2022-09-16

**Authors:** Yue Xu, Xuliang Zhang, Xiujun Liu, Zhaohui Zhang

**Affiliations:** 1 School of Tourism and Geography Science, Qingdao University, Qingdao 266071, China; 2 The First Institute of Oceanography, Ministry of Natural Resources, Qingdao 266061, China

**Keywords:** national wetland park, spontaneous vegetation, diversity, flowering season, habitat

## Abstract

Spontaneous vegetation plays an important role in protecting urban biodiversity and the maintenance of urban ecosystems. In this study, we investigated the species diversity, life-form composition, origin, flowering season, and spatiotemporal distribution of spontaneous vegetation in the exhibition and education area of Tangdao Bay National Wetland Park using the quadrat survey method. There were 65 spontaneous association types and 210 spontaneous plant species, belonging to 151 genera and 44 families. The associations and species of spontaneous plants in roadside habitats were found to be the highest. In addition, many species were found in woodland and lakeside habitats, whereas the lowest number of species were found in coastal habitats. The life-form composition included 76 annual and 94 perennial herb species. These plants were of various origins. There were 160 native, 9 domestically introduced, 2 introduced alien, and 39 invasive alien plant species, which predominantly came from the Americas. A single peak was observed from March to November for the spontaneous plant species that were in their growing season, including those of different life forms and from various sources. The same was true for spontaneous plants in their flowering season. During their growing season, the number of spontaneous plant species was highest during September and, during their flowering season, the number of species was highest in July. From April to September, the Shannon–Wiener diversity index for spontaneous vegetation in the roadside habitat was the highest, followed by those for the woodland and lakeside habitats, and that of the coastline habitat was the lowest. The monthly average Shannon–Wiener diversity index for spontaneous plant associations in the four habitats also varied, with a single peak. The diversity of spontaneous plants and alien invasive plants in Tangdao Bay National Wetland Park is high. The wise use and protection of spontaneous flowering plants with long ornamental seasons can effectively reduce the maintenance costs, resource consumption, and energy requirements of the park. Spontaneous plants should also be managed to reduce the harm from alien invasive plants in the park, and alien invasive plants should be removed from the park during their flowering seasons.

## 1. Introduction

The rapid process of urbanisation has had considerable negative impacts on natural ecosystems, and this has caused continuous urban environmental problems. The need to maintain the functions of urban ecosystems and improve human well-being has attracted considerable attention [1,2]. To improve the ornamental value of urban ecological landscapes, urban areas that incorporate green spaces are currently being designed. These include park, residential, transportation, and garden green spaces. However, most of these efforts have resulted in landscape homogeneity, a decline in the species diversity of plant communities, an imbalance in interspecific relationships, loss of animal habitats, and high construction and maintenance costs [3,4]. Spontaneous plants in urban green spaces have previously been regarded as targets for weed control [5]. As human ecological consciousness has gradually improved, landscape designers have begun to pay attention not only to the species composition of green plants but also to natural spontaneous vegetation populations, especially those with high and species diversity, strong adaptability, and a wide distribution, when planning and designing urban green spaces. They have now realised that the cost of maintaining and managing spontaneous vegetation is relatively low. Spontaneous vegetation is very important in the context of constructing unique urban landscapes [6,7] to maintain urban ecosystem services for soil and water conservation, protect insect and bird biodiversity [8,9], and purify polluted soil [10,11]. However, there is a competitive relationship between spontaneous self-reproductive vegetation and planted greening plants.

There is a long history of research on spontaneous vegetation [12]. However, research on spontaneous vegetation in urban green spaces in China is still in its early stages. Research on spontaneous vegetation, including species composition, spatiotemporal distribution, interspecies relationships, and how it helps maintain biodiversity, should be expanded. In addition, the plant community needed to maintain the stability of urban ecosystem should be further studied [13]. The construction of national wetland parks is important for the protection and wise use of wetlands in China, and has become more widespread with time. Between 2005 and 2020, 899 national wetland parks with an area of at least 100 hm^2^ were designated. These helped expand urban green spaces and effectively protected spontaneous vegetation in some cities. However, constructing them also contributed to an increase in the number of alien invasive plant species in urban areas. Tangdao Bay National Wetland Park, Qingdao City, China, is well-constructed as a destination for tourists and is also a suitable urban green space for locals to relax. Research on the habitat characteristics, species composition, spatiotemporal distribution, plant sources, and biodiversity of spontaneous vegetation in urban green spaces, including national wetland parks, is very important. This information can assist in the construction of landscapes in urban green spaces that incorporate spontaneous vegetation, which can alleviate the loss of natural landscapes in urban areas, improve biodiversity, prevent the invasion of harmful alien invasive plants, satisfy urban residents’ desire for ornamental flowering plants, and help to maintain urban biodiversity and the stability of urban ecosystems [12,14,15].

## 2. Materials and Methods

### 2.1. Study Region

**Figure 1:**
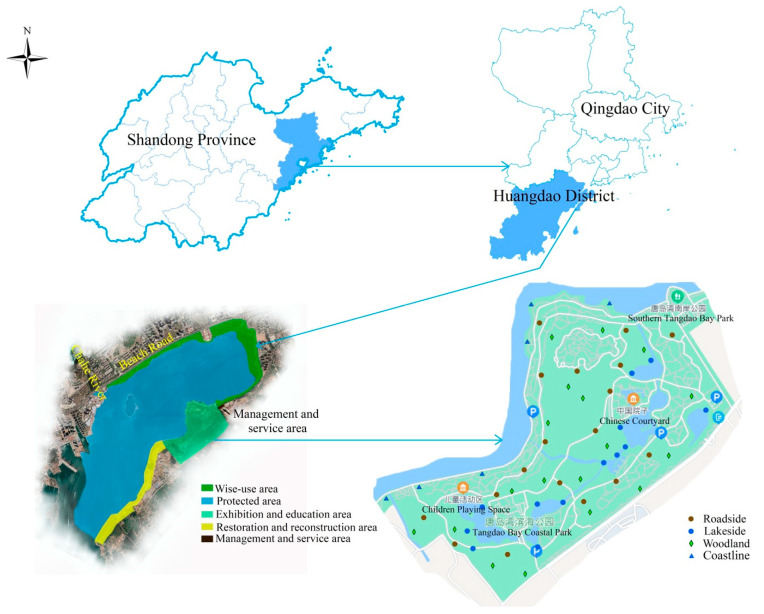
Study area and locations of sampling quadrats.

Tangdao Bay is located on the southwest side of Jiaozhou Bay, Huangdao District, Qingdao City, Shandong Province, China (Figure 1). The bay is a primary tectonic bay with a long axis in the northwest–southeast direction. It has a length of 7.8 km, and its width at the bay mouth is 2.6 km. The opening of Tangdao Bay to the southwest is approximately 17 km^2^ in area. There are three islands in the bay, namely, Tangdao, Niudao, and Jidao. The annual average sea surface temperature is 13.5 °C, and the tide for Tangdao Bay is a regular semidiurnal tide with an average tidal range of 2.7 m and a maximum tidal range of 3.5 m [16,17]. Most of the Tangdao Bay coastline is rocky, with gravel beaches below the sea cliffs and low hills along the coast. The main soil-forming parent materials are slope alluvium, plain alluvium, and diluvium, and the most common soils are brown and fluvo-aquic. The climate is that of a temperate monsoon zone, with warm winters, cool summers, abundant rainfall, distinct seasons, a perennial mean temperature of 12.5 °C, and an annual precipitation of 750 mm. There are two short seasonal rivers with rapid runoff, namely, the Chahe River and the Zhoujiakuang River, which flow into the bay from the north.

The Tangdao Bay National Wetland Park (120°09′59″−120°19′49″ E, 35°54′10″−35°55′58″ N) comprises the waters of the bay, the tidal flats, and a buffer area along the coast, with a width of 50–100 m. The spatial area of the park is 1637.6 hm^2^, of which the wetland area is 1312.8 hm^2^. The wetland area accounts for 80.2% of the total area of the park. The area encompasses offshore and coastal wetlands, artificial wetlands, and seven wetland types, including shallow-water areas, sandy beaches, muddy beaches, estuaries, reservoirs, ponds, canals, and aquaculture farms. A compound bay-island-coastal ecosystem with a high ecosystem service value is formed by the seven wetland types [18]. The primary natural vegetation is composed of temperate deciduous broad-leaved trees, and the park is dominated by various species of halophytic, marsh, aquatic, and sandy vegetation.

### 2.2. Research Methods

To study the biodiversity and spatiotemporal distribution of the spontaneous vegetation in Tangdao Bay National Wetland Park, a ring survey line was set up along the main tourism roads, with quadrats in the roadside, woodland, lakeside, and coastline habitats in the exhibition and educational areas of the park. The spontaneous vegetation in these quadrats, which was surveyed monthly between March and November. Only spontaneous plant species were surveyed and, during the growth season, there were no quadrats with only a few spontaneous plants in March, October, and November. Each quadrat had an area of 1 m^2^. The number of species, individuals, and species in their flowering season in each quadrat were surveyed and recorded each month, as was the area of the canopy cover formed by vegetation associations. A total of 16, 27, 24, 55, 28, and 41 quadrats were surveyed from April to September, and the positions of most of the quadrats were the same in different months.

The spontaneous plant species were determined according to the descriptions in the *Flora of China* (visit http://www.iplant.cn/foc (accessed on 1 July 2022)), and the *Plant Photo Bank of China* (visit http://ppbc.iplant.cn/ (accessed on 1 July 2022)). The certified spontaneous plants were divided into four types: native, introduced domestic, introduced alien, and invasive alien plants. Native plants were defined as spontaneous plants with a natural distribution in the area, including Qingdao City, whereas the introduced domestic and alien plants were defined as those intentionally or unintentionally introduced from other areas in China. Alien invasive plants were defined as plants intentionally or unintentionally introduced from other countries worldwide, leading to the establishment of self-reproducing populations through naturalisation. Colonisation by invasive alien species can affect and destroy the native ecology, leading to economic impacts or habitat loss [19,20]. During the survey, the number of spontaneous plant species in the quadrats was counted, and the canopy cover was estimated. Spontaneous plants were divided into three types of species, namely, constructive, dominant, and companion species, depending on their richness, average height, and canopy cover.

Based on this survey, the vegetation association types, number of spontaneous plant species, composition of family, genera, and species, spatiotemporal distribution, life forms, flowering seasons, distribution frequency in different habitats, and number of native plant species, domestically introduced plants, and alien invasive plants were examined. In addition, the grades of alien invasive plants, simplified dominance index, and diversity index of vegetation associations in the different habitats studied were analysed. The number of alien invasive plant species in the park that were spontaneous plants was compared with that of the Hangzhou Xixi National Wetland Park, Zhejiang Province, and some other wetlands in China. Some measures have been proposed to construct landscapes for flower appreciation and to defend against alien plant invasion. The following formulas were used to calculate the distribution frequency of the spontaneous plant species, the simplified dominance index, and the Shannon–Wiener diversity index of spontaneous vegetation:

The distribution frequency of spontaneous plants (*F*) is defined as:

*F* = (*S_i_*/*N*) × 100% (1)

where *S_i_* is the quadrat number of species *i* and its distribution, and *N* is the total number of quadrats surveyed during each month [21].

The simplified dominance for spontaneous vegetation (*Sd_i_*) is defined as:

*Sd_i_* = (*C_i_* × 100 + *A_i_*)/2 (2)

where *Sd_i_* is the simplified dominance of spontaneous plant species *i*, *C_i_* is the canopy cover of spontaneous plant species *i* in the quadrat (i.e., the ratio of the vertical projection area of the above-ground part of species *i* to the total area of the quadrat), and *A_i_* is the number of individuals of species *i* in the quadrat.

The Shannon–Wiener diversity index (*H*) is defined as:

*H* = − Σ*P_i_*ln*P_i_* (3)

where *P_i_* is the proportion of the simplified dominance of species *i* of a spontaneous plant to the sum of the simplification dominance of all spontaneous plants in the quadrat.

## 3. Results

### 3.1. The Associations and Composition of Family, Genera and Species of the Spontaneous Vegetation

#### 3.1.1. The Association Types of the Spontaneous Vegetation

When we examined the associations among the constructive species in the park, we found 65 association types, including Ass. *Phragmites australis* and Ass. *Suaeda salsa*. The number of roadside, lakeside, woodland, and coastline habitats in the park was 37, 27, 25, and 18, respectively (Table 1). The association types along the roadside were the highest but most disturbed by human activities. The lowest number of association types was found on the coastline in areas with coarser, infertile, and saline soil. Ass. *Setaria viridis*, Ass. *Erigeron annuus*, Ass. *Artemisia argyi*, Ass. *Imperata cylinders* were the most widely distributed associations, and the distribution frequency of Ass. *Setaria viridis*, Ass. *Erigeron annuus*, and ass. *Digitaria sanguinalis* was the most abundant among all the associations.

#### 3.1.2. The Composition of Families, Genera, and Species of the Spontaneous Plants

There were 210 spontaneous plant species belonging to 151 genera and 44 families in the exhibition and education areas of the national wetland park (Supplementary Table S1). This included nine relatively large families containing over five species, including Asteraceae (which has 24 genera/43 species), Gramineae (29/32), Leguminosae (11/15), Brassicaceae (9/13), Polygonaceae (2/9), Caryophyllaceae (6/8), Rosaceae (6/8), Cyperaceae (4/7), and Chenopodiaceae (3/6). There were 141 species of spontaneous plants from 94 genera in these families, and the number of spontaneous plants in these families, genera, and species accounted for 20.45%, 66.25%, and 67.14% of the total number recorded in the park. There were six relatively large genera containing over four species: *Artemisia*, with eight species; *Persicaria*, with six species; *Ixeridium*, with five species; and *Sonchus*, *Bidens*, and *Chenopodium*, with four species each. There were 31 species of spontaneous plants in these genera, and the number of spontaneous plant species in these two relatively large genera accounted for 3.97% and 14.76% of the total, respectively. There were 18 families that comprised a single genus and a single species, including the families *Oxalis pes—caprae*, *Euonymus fortunei*, and *Phytolacca acinosa*. Families containing a single genus and a single species accounted for 40.91%, 11.92%, respectively, and 8.57% of the total recorded in the park. The presence of species of relatively large families and relatively large genera, and the presence of species belonging to families containing only one genus and one species, indicate that the differentiation of families is relatively low and the differentiation of genera is relatively high. There were many relatively large families with many species, and a few relatively large genera with many species.

#### 3.1.3. Composition of Families, Genera, and Species of Spontaneous Vegetation in Different Habitats

**Figure 2:**
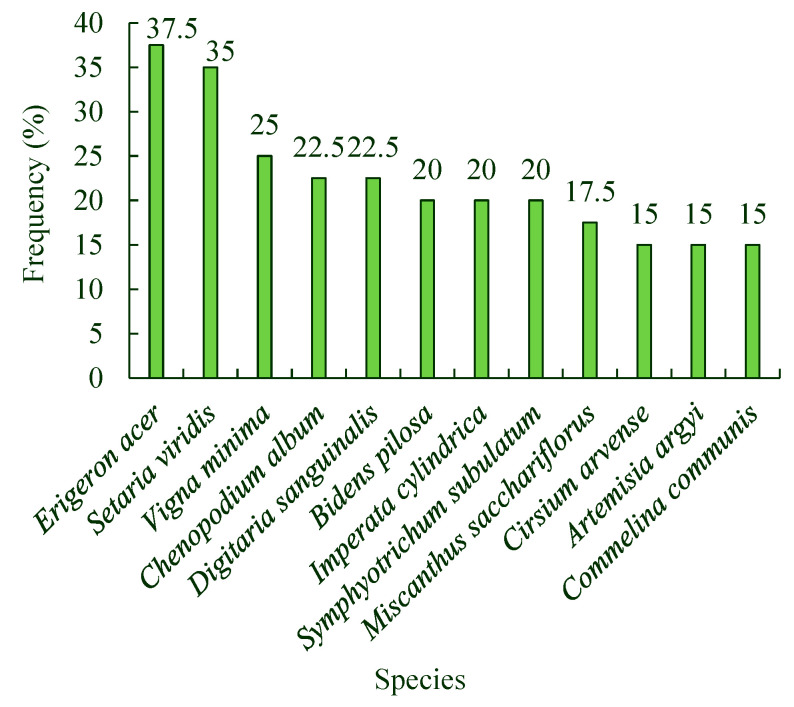
Distribution frequency of the 12 most widely distributed species of spontaneous plants in the Tangdao Bay National Wetland Park.

There are four habitat types in exhibition and education areas: roadside, lakeside, woodland, and coastal. There were 149 species of spontaneous plants belonging to 105 genera and 33 families in the roadside habitat. There were 28 families in the lakeside habitat, including 81 species from 63 genera. There were 32 families in the woodland habitat, including 80 species from 63 genera. Finally, there were 27 species from 22 genera and 12 families in the coastal habitat. Members of the families Asteraceae, Gramineae, Polygonaceae, Caryophyllaceae, Rosaceae, Chenopodiaeae, Plantaginaceae, Scrophulariaceae, Boraginaceae, and Labiatae were the most widely distributed spontaneously occurring plants (Table 2). There were 12 most widely distributed species of spontaneous plants in the exhibition and educational area of the park, with a distribution frequency of 15–37.5% (Figure 2).

### 3.2. The Life Form Composition of Spontaneous Plants

**Figure 3:**
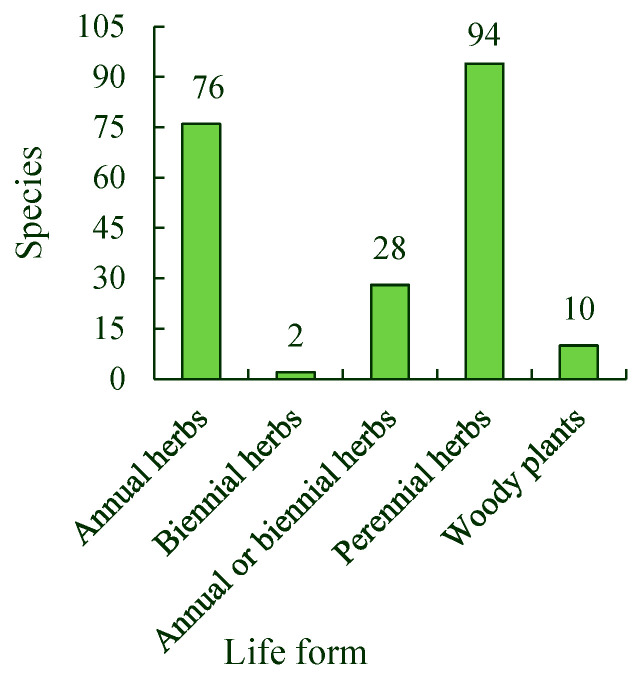
Spontaneous plant species in different life forms.

The spontaneous plants in the park were divided into five life-form types: annual, biennial, annual or biennial, and perennial herbs, and woody plants. There are many species of annual and perennial herbs, and some of these species were spontaneous plants that appeared in the park. There were 76 annual herb species, including *Artemisia capillaris*, *Xanthium sibiricum*, and *Digitaria sanguinalis*. There were two biennial herb species: *Erigeron acer* and *Stellaria uliginosa*. There were 28 species of annual or biennial herbs, including *Silene aprica* and *Veronica persica*. There were 94 perennial herb species, including *Cynodon dactylon*, *Ixeridium gramineum*, and *Zoysia japonica*. There were also 10 species of woody plants, comprising four tree species, four shrub species, and two species that can exist either as trees or shrubs. These species included *Paulowinia fortunei*, *Ulmus pumila*, and *Tamarix chinensis.* The proportions of annual herbs, biennial herbs, annual or biennial herbs, perennial herbs, and woody plants to the total species of spontaneous plants were found to be 36.19%, 0.95%, 13.33%, 44.76%, and 4.76%, respectively (Figure 3).

### 3.3. The Sources of Spontaneous Plants, and Invasion Sources, Invasion Levels and Life Forms of Alien Invasive Plants

#### 3.3.1. Sources of Spontaneous Plants

**Figure 4:**
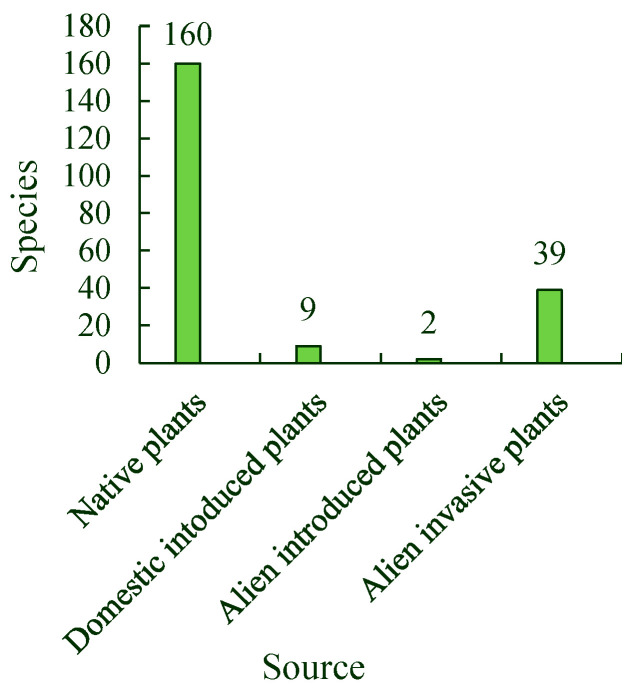
Spontaneous plant species from different sources.

Depending on their source, spontaneous plants can be divided into four types: native plants, domestically introduced plants, alien introduced plants, and alien invasive plants. There were 160 native plant species, including *Plantago asiatica*, *Ixeridium chinense*, and *Taraxacum mongolicum*. There were nine species of domestically introduced plants, including *Cynodon dactylon*, *Lagedium sibiricum*, and *Stellaria uliginosa*, and two species of alien introduced plants, *Ixeridium sonchifolia* and *Oxalis pes-caprae*. There were also 39 species of alien invasive plants, including *Erigeron annuus*, *Galinsoga parviflora*, *Trifolium repens*, and *Ricinus communis* in the park (Figure 4). The proportions of native, domestically introduced, alien introduced, and alien invasive plant species comprising the total number of spontaneous plant species were 76.19%, 4.29%, 0.95%, and 18.57%, respectively.

#### 3.3.2. Alien Invasive Plant Grades

Alien invasive plants can be divided into five grades depending on the scope of their invasion and the ecological or economic losses caused by their invasion. Grade 1 malignant alien invasive plants are those with an invasive scope of more than one geographic area that have caused vast ecological or economic losses at the national level. Grade 2 alien invasive plants are those with an invasive scope of at least one geographic area that have caused relatively large ecological or economic losses at the national level. Local grade 3 alien invasive plants are those with an invasive scope of at least one geographic area and have caused regional ecological or economic losses. The general alien invasive plants in grade 4 are those without pronounced ecological or economic losses and with a recent invasion trend, regardless of whether their invasive scopes are wide or narrow. Alien invasive plants in grade 5 are those that have been newly reported and insufficiently studied. They, have a short occurrence time, and their invasion trends have not yet been determined [21,22,23]. There are 6, 11, 7, 14 and 1 species of alien invasive plants in grades 1, 2, 3, 4 and 5, respectively, in the exhibition and education area of the national wetland park.

#### 3.3.3. The Sources, Life Forms, and Distribution of Alien Invasive Plants

The 39 alien invasive plant species found in the exhibition and education area of Tangdao Bay National Wetland Park belonged to 35 genera and 16 families, among which 10 genera and 12 species belonged to the Asteraceae family. The proportion of families, genera, and species of alien invasive plants to those of all spontaneous plants were 36.36%, 23.18%, and 18.57%, respectively. The alien invasive plants have many sources, including 21 species from the Americas, one species from Europe and Central Asia, one species from West Asia and Euorpe, seven species from Europe, two species from Africa, three species from West Asia, three species from India, and one species of uncertain origin. When classified by life form, there were 23 species of annual herbs, 7 species of annual or biennial herbs, 8 species of perennial herbs, and 1 species of woody plant (a shrub), which accounted for 58.97%, 17.95%, 20.51%, and 2.56% of the total species, respectively (Table 3). There were 29, 13, 11, and 2 species of alien invasive plants in roadside, lakeside, woodland, and coastal habitats, respectively. They accounted for 74.36%, 33.33%, 28.21%, and 5.13% of the total number of alien invasive plant species, respectively. *Erigeron* *annuus* was found to be the most widely distributed species in the four habitats, followed by *Trifolium repens*, *Bidens pilosa*, *Bidens bipinnata*, *Veronica persica*, and *Chenopodium serotinum.*

### 3.4. The Spatiotemporal Changes in the Total Species, Sources, and Life Forms of Spontaneous Plants

#### 3.4.1. The Spatiotemporal Change in the Total Number of Spontaneous Plant Species

**Figure 5:**
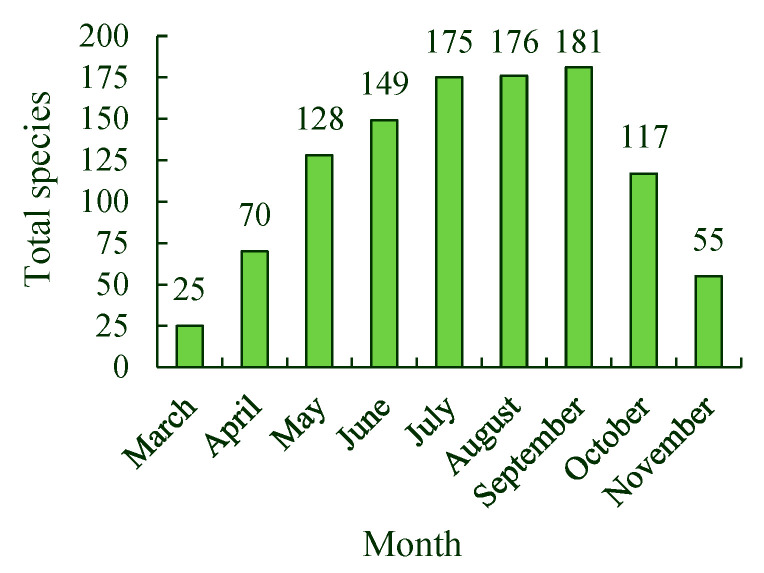
Number of spontaneous plant species present during the growing season, from March to November.

The total number of spontaneous plant species in Tangdao Bay National Wetland Park first increased and then decreased from March to November, and a single peak was observed. There were 25 species of spontaneous plants belonging to 24 genera and 10 families in the growing season in March, the number of spontaneous plant species increased to a peak of 181 species from 133 genera and 42 families in September. The spontaneous plants gradually became quiescent in October. In November, at the end of the growing season, 55 species and 49 genera from 24 families were observed (Figure 5).

Spontaneous plants in Asteraceae, Gramineae, Leguminosae, and Brassicaceae were more common than those in other families throughout the growing season from March to November. The largest number of spontaneous plant species throughout the year was members of Asteraceae, and 38 species in this family were observed in July. The number of species belonging to Gramineae and Leguminosae families was 29 and 15 in September, respectively. The number of spontaneous plant species in Brassicaceae varied between 9 and 11 from May to September.

There were pronounced differences in the numbers of spontaneous plant species in the different habitats that were studied. The number of spontaneous plant species was the highest in the roadside habitat, and there were also many species in the lakeside and woodland habitats. The number of species was the least abundant in the coastline habitat during the entire growing season. Each habitat contained 150, 82, 80, and 24 species, respectively. In September, the number of spontaneous plant species in the roadside, lakeside, woodland, and coastline habitats were 127, 81, 75, 76, and 21, respectively. These were the highest numbers recorded during the year (Table 4).

#### 3.4.2. The Spatiotemporal Changes in Species of Spontaneous Plants from Different Sources

**Figure 6:**
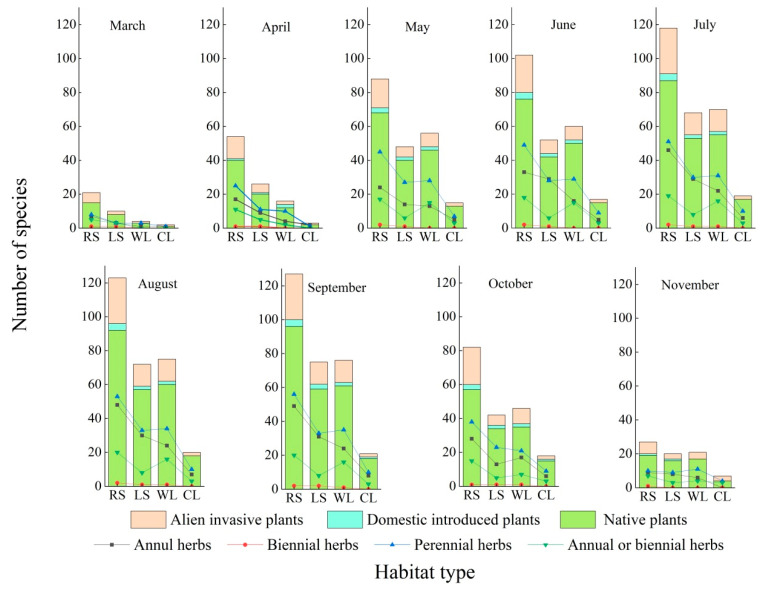
Spatiotemporal variations in species of spontaneous plants from different sources and in different life forms. RS—roadside, LS—lakeside, WL—woodland, CL—coastline.

The number of native, domestically introduced, and alien invasive plant species in the roadside, lakeside, woodland, and coastline habitats initially increased and then decreased, with a single peak during the growing season. The number of native plant species in the roadside, lakeside, and woodland habitats increased from 15, 8, and 3 to 96, 59, and 61, respectively, from March to September. From September to November, the number decreased to 19, 16, and 17, respectively. The number of native plant species in the coastline habitat increased from one to eighteen from March to August and decreased from eighteen to four from August to November. In the roadside habitat, domestically introduced and alien invasive plant species were most common, while in the lakeside and woodland habitats they were less common, and in the coastline habitat from May to September, they were least common. The total number of domestically introduced and alien invasive plant species in the roadside habitat was 31, and they appeared most in July and August. The number of species in the lakeside habitat was 16, and they were most common from July to September. The number of species in the woodland habitat was 14 and was the highest in July. Three species were the most common in the coastline habitat in September and October. The difference in native plant species among the four habitats was the largest, and that of domestically introduced plant species was also high. The least difference was observed in the alien invasive plant species. The differences in the native, domestically introduced, and alien invasive plant species were the highest in the roadside habitat and the lowest in the coastline habitat. The differences in the lakeside and woodland habitats were in between those of the roadside and coastline habitats. The range of seasonal species change was the highest for the native plant species in all four habitats was the largest, and the next highest range was for the domestically introduced plant species. The lowest range was recorded for the alien invasive plant species (Figure 6).

**Figure 7:**
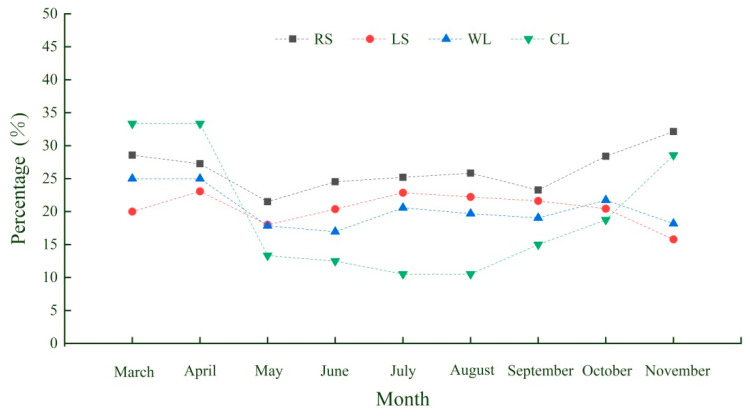
Spatiotemporal variations in the proportion of alien invasive plant species to all spontaneous plant species.

From March to November, the proportion of domestically introduced and alien invasive plant species to the total species of spontaneous plants in the four habitats varied between 10.53% and 33.33%. The average proportion of domestically introduced and alien invasive plant species to the total species of spontaneous plants in the four habitats was the highest (27.17%) in April, and the proportion was the highest in the coastline habitat, followed by those of the roadside and woodland habitats, and along the coastline. From May to September, the proportion of domestically introduced and alien invasive plant species to all the species of spontaneous plants in the roadside habitat was the highest, followed by those of the woodland and lakeside habitats, and was the lowest in the coastline habitat. The proportion was the highest in the roadside habitat, followed by those of woodland and lakeside habitats, and was the lowest in the coastline habitat during October. The proportion was the highest in the roadside habitat, followed by the coastline and woodland habitats, and was the lowest in the lakeside habitat in November (Figure 7).

#### 3.4.3. The Spatiotemporal Change in Spontaneous Plant Species Depending on Life Form

Among the four habitat types, the number of annual and biennial herb species was the highest in the roadside habitat, and was lower in lakeside and woodland habitats. The coastline habitat had the lowest number of these species. Perennial herb, woody plant, and annual or biennial herb species were the most common in the roadside habitat, were less common in the woodland and lakeside habitats, and were the least common in the coastline habitat (Figure 6).

The spontaneous plant species from different life forms initially increased and then decreased from March to November in all four habitats, and a single peak was observed. The species peaks for annual herbs in the roadside, lakeside, and coastline habitats occurred in September, and were 49, 31, and 8, respectively. The species peak for annual herbs in the woodland habitats was 24 and occurred in August. The temporal changes in the biennial herb species were relatively low in all four habitats for a few biennial herb species, with peaks in the roadside, lakeside, and woodland habitats at 2, 2, and 1, respectively. No biennial herbs were recorded along the coastline. In September, the species peaks for perennial herbs in the roadside and woodland habitats were 56 and 35, respectively, and there were 33 species in the lakeside habitat during August and September. The total number of perennial herb and woody plant species was 10 and was the highest in the coastline habitat in July, August, and September. The number of annual or biennial herb species in the roadside habitat reached a peak of 20 in August and September, whereas the number of species in the woodland and lakeside habitats were 16 and 8, respectively, during July, August, and September. The species in the coastline habitat changed slightly and remained at three species from May to September (Figure 6).

### 3.5. The Changes in Flowering Season of the Spontaneous Plant Species

**Figure 8:**
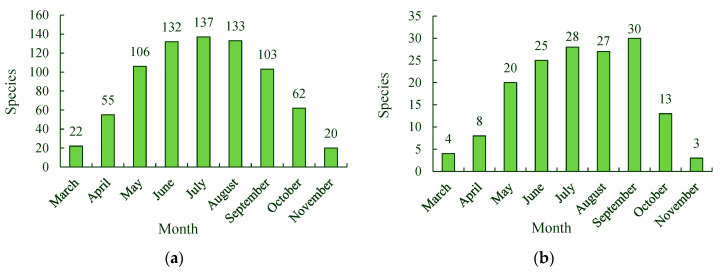
Spontaneous and alien invasive plant species present in their flowering seasons from March to November. (**a**) Species of spontaneous plants in their flowering season. (**b**) Species of alien invasive plants in their flowering season.

Spontaneous plant species were present during the flowering season in Tangdao Bay National Wetland Park, from March to November. There were 22 spontaneous plant species present in their flowering seasons in March, including four species of alien invasive plants, namely, *Veronica persica*, *Bidens bipinnata*, *Coronopus didymus*, and *Ceratium glomeratum*. The number of spontaneous plant species present during their flowering seasons increased to 55 in April and included eight alien invasive plants species, such as *Lepidium virginicum*, *Capsella bursa-pastoris*, *Chenopodium serotinum*, *Euphorbia maculata*, and *Geranium carolinianum*. The number of species increased to 106 in their flowering seasons in May and included 20 species of alien invasive plants such as *Sonchus oleraceus* and *Conyza bonariensis*. In June, July, and August, there were 132, 137, and 133 spontaneous plant species present during their flowering seasons, including 25, 28, and 27 alien invasive plant species, respectively. Eight species of alien invasive plants, including *Erigeron annuus*, *Aster subulatus*, *Eclipta prostrata*, and *Chloris virgata*, began their flowering seasons in June. Four species of alien invasive plants, namely, *Galinsoga parviflora*, *Chenopodium glaucum*, *Amaranthus retroflexus*, and *Gaura parviflora*, entered their flowering season in July. The alien invasive plant, *Helianthus tuberosus*, entered its flowering season in August. The number of spontaneous plant species in their flowering season rapidly decreased after August. The spontaneous plant species present in September and October were 103 and 62, respectively, and included, 20 and 13 alien invasive plant species, respectively. There were 20 spontaneous plant species, such as *Artemisia annua* and *Cirsium japonicum*, and three species of alien invasive plants, namely, *Cyperus rotundus*, *Sonchus oleraceus*, and *Solidago canadensis*, present during their flowering season in November (Figure 8).

### 3.6. The Spatiotemporal Changes in the Shannon–Wiener Diversity Index of Spontaneous Associations

**Figure 9:**
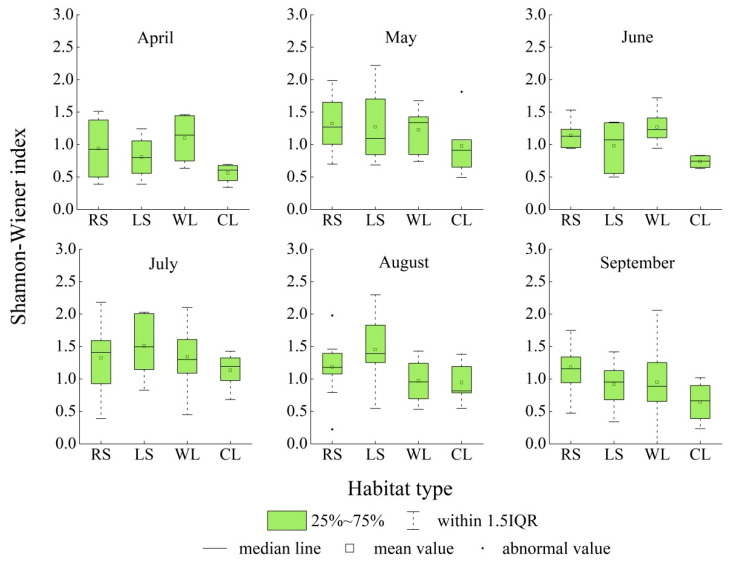
Range of variation and monthly medians for the Shannon–Wiener diversity index in different habitats of the Tangdao Bay National Wetland Park from April to September.

The monthly average Shannon–Wiener diversity index for spontaneous associations in the roadside habitat was the highest, followed by woodland and lakeside habitats, and the coastline habitat had the lowest index from April to September. The average values for the monthly Shannon–Wiener diversity index of spontaneous associations in the roadside, woodland, lakeside, and coastline habitats were 1.1819, 1.1567, 1.1425, and 0.8333, respectively. The monthly average Shannon–Wiener diversity index for spontaneous associations in the four habitats first increased and then decreased, with single peaks in July. The ranges of variation were 0.9393–1.3239, 0.8080–1.5085, 0.9507–1.3404, and 0.6458–1.3320, respectively. The range of variation for the monthly average Shannon–Wiener diversity index for spontaneous associations in the lakeside habitat was the highest among the four habitat types. The average median values for the monthly Shannon–Wiener diversity index for spontaneous associations in the roadside, woodland, lakeside, and coastline habitats were 1.1783, 1.1433, 1.1343, and 0.8229, respectively. The median value for the monthly Shannon–Wiener diversity index of spontaneous associations in the roadside, lakeside, and coastline habitats first increased and then decreased, with single peaks of 1.409, 1.497, and 1.194 in July. The median value for the monthly Shannon–Wiener diversity index of spontaneous associations in woodland habitats decreased from April to September, with the largest median value of 1.146 in April (Figure 9).

## 4. Discussion

### 4.1. The Diversity of Spontaneous Plants in the Tangdao Bay National Wetland Park Is High

There were 210 spontaneous plant species from 151 genera and 44 families recorded in the Tangdao Bay National Wetland Park. There were 260 species of spontaneous plants from 214 genera and 82 families in Hangzhou Xixi National Wetland Park [24]. A total of 107 spontaneous plant species from 89 genera and 32 families were recorded in the central urban area inside the outer ring road of Shanghai City [25], and 127 species of spontaneous plants from 101 genera and 48 families were recorded in the urban area of Ningbo City, China [26]. These three cities are in subtropical monsoon climate zones. There were 128 species of spontaneous plants from 98 genera and 32 families recorded in the Olympic Forest Park of Beijing City, which is also in a temperate monsoon climate zone [14]. There were 95 species of spontaneous plants from 75 genera and 37 families recorded in the Xian City urban area, which is located in a temperate semiarid climate zone on the Loess Plateau in China [27]. There were substantial differences in the species of spontaneous plants in different habitats, and significant changes in spontaneous plant species during the growing season from March to November. As there are relatively few woody spontaneous plant species, and all of them can be observed and identified every month, seasonal variations in woody spontaneous plant species in different habitats during the growing season were not analysed.

The population of spontaneous plant species in urban built-up areas is influenced by climate, urbanisation level, the construction of urban green spaces, and other factors. The species and diversity of spontaneous plants in the Tangdao Bay National Wetland Park were higher than those in the Olympic Forest Park of Beijing City, as well as those in the urban built-up areas of Xian City, Shanghai City, and Ningbo City. The results indicate that the original natural habitats in the Tangdao Bay National Wetland Park were not strongly affected by urbanisation, as indicated by studying four habitats in the park. However, the native vegetation vanished, which was an important habitat for spontaneous plant species in the urban built-up area of Qingdao City. The national wetland park and its spontaneous vegetation play an important role in protecting the urban biodiversity of Qingdao City. Therefore, the park’s spontaneous vegetation should be protected.

### 4.2. There Were Many Species of Alien Invasive Plants of Spontaneous Plants in the Park

The spontaneous plant population of Tangdao Bay National Wetland Park includes 39 species of alien invasive plants from 35 genera and 16 families. For comparison, 27 alien invasive plant species belonging to 21 genera and 13 families were recorded in the Dahuangpu Provincial Nature Reserve of Tianjin City, China [28]. In addition, there were 39 species of alien invasive plants belonging to 26 genera and 14 families recorded in the wetlands of Sishui County, Ji’ning City, Shandong Province and 49 species of alien invasive plants belonging to 35 genera and 18 families in the wetlands of Luoyang City, Henan Province. These wetlands are all located in temperate zones. There were 55 species of alien invasive plants from 43 genera and 22 families (55/43/22), 69/52/27, 30/27/20, 51/41/20, 36/32/20, 24/20/15 and 15/13/8, respectively, in the wetlands, which are located in the subtropical zone (Table 5) [29,30,31].

There are many species of alien invasive plants in the exhibition and education area of Tangdao Bay National Wetland Park, which has an area of only 173.3 hm^2^. These species were especially abundant in the roadside habitats of the park. Some species of alien invasive plants quickly breed and enlarge their distribution area, occupy the ecological niches of native species, decrease the ornamental value of ecological landscapes, influence biodiversity protection, and may contribute to potential adverse impacts over a large area around the park. For example, *Erigeron annuus* limits the growth of native spontaneous plants and decreases regional biodiversity by allelopathy, producing between 1 × 10^4^ and 5 × 10^4^ mature seeds per plant in a single growing season. The seeds are quickly dispersed by the wind and seriously endanger the crops and fruit trees around the park [32,33]. The invasion of *Solidago canadensis* also alters the physical and chemical properties of soil, influences soil respiration, and increases soil moisture content and carbon loss. All these changes affect the community structure of soil animals and lead to a decline in the number of species and individuals [34,35]. A few species of alien invasive plants, such as *Bidens pilosa*, also produce thorny seeds which can easily penetrate and attach to the clothes of tourists and residents, making them feel uncomfortable and less likely to be attracted to the park.

### 4.3. Creating Diverse Landscapes with Spontaneous Plants and Removing Malignant Alien Invasive Plants According Their Flowering Season

Spontaneous plant species were present in the park during their flowering seasons every month from March to November; however, the monthly number of spontaneous plant species during the flowering season varied considerably. In July, the number of spontaneous plant species in the park during the flowering season was the highest, with 137 species. In November, the number of spontaneous plant species present in the park during the flowering season was the lowest, with 20 species. Spontaneous plants with strong adaptability and good growth characteristics can be used based on their flowering seasons to create diverse landscapes with long ornamental seasons in different habitats to reduce the cost of maintenance and the consumption of resources and energy required to manage landscapes within the park.

There were more spontaneous species in the Tangdao Bay National Wetland Park of Qingdao City and the Xixi National Wetland Park in Hangzhou City than in other wetlands in China. This indicates that the construction of national wetland parks has resulted in an increase in alien invasive plant species in wetlands and in the surrounding areas affected by human activities. Native spontaneous plants should be adequately used and effective measures should be implemented to reduce the harm of malignant alien invasive plants to wetlands, appropriately introduce alien plants, and prevent invasion by alien invasive plants during the construction of wetland parks in the future.

The monthly changes in the alien invasive plant species during the flowering season were roughly the same as those of the species of all spontaneous plants from March to November. The species of alien invasive plants during the flowering season were more abundant from May to September than in other months, and tourists could be guided to recognize the harmful alien invasive plants based on when these plants flower. This would improve their awareness of their natural environment and the steps needed to protect it. The malignant alien invasive plants in grade 1, which cause serious damage to the regional ecosystem, enter their flowering seasons in different months. *Pharbitis purpurea* enters its flowering season in May, *Erigeron annuus* and *Aster subulatus* enter their flowering seasons in June, *Amaranthus retroflexus* enters its flowering season in July, *Bidens pilosa* enters its flowering season in August, and *Solidago canadensis* enters its flowering season in October. These alien invasive plants should be removed before or at the beginning of their flowering seasons to reduce their negative impacts on wetland ecosystems and humans.

### 4.4. The Research Results Are Useful for the Development of Interdisciplinary Thematic Learning Strategies for Junior High Schools near Tangdao Bay National Wetland Park

The Ministry of Education of the People’s Republic of China published the Geography Curriculum Standards for Compulsory Education (2022 edition) in March 2022. The curriculum standards added content for teaching interdisciplinary thematic learning in wetlands research, suggesting that junior high schools could implement this guidance through using local National Wetland Parks [36]. Thus, our research will also be helpful to the teachers and students of hundreds of junior high schools. The required topics include drawing electronic maps of the wetland park, recognizing various wetland plants, exploring wetland ecosystem services, and making rational proposals for the wise use and protection of wetlands.

### 4.5. The Value and Limitations of This Research

This research enhanced the understanding of spontaneous plant diversity in the national wetland park and urban green space of Qingdao City, and can also be used to provide effective guidance for administrators, such as creating diverse landscapes of spontaneous flowering plants with long ornamental seasons, goosing malignant alien invasive plants according to their flowering season to reduce maintenance costs and the consumption of energy and resources by the wetland park, developing strategies for science popularisation and improving the publicity of the park as well as the protection of spontaneous plant diversity of urban green space in Qingdao City. This information can also be used in the design and construction of new national wetland parks.

However, this research does have some limitations. For example, the survey area only included the exhibition and education area of the park, which is relatively small. Studying a larger area would be the best way to gain a deeper understanding of the biodiversity and spatiotemporal changes in spontaneous plant species. In addition, research on the invasion tendencies, harm, and mechanisms of alien invasive plants should be investigated in more detail.

## 5. Conclusions

The Tangdao Bay National Wetland Park in Qingdao City has a high diversity of spontaneous plants. The associations and species of spontaneous plants in the roadside habitat were the highest, followed by those in the woodland and lakeside habitats, and those along the coastline were the lowest. Twelve species of spontaneous plants, including *Erigeron acer*, *Setaria viridis*, *Chenopodium album*, *Artemisia argyi*, and *Commelina communis*, were the most widely distributed. When classified by life form, spontaneous plants were found to be mainly annual and perennial herbs. The spontaneous plants were divided into four types: native plants, domestically introduced plants, alien introduced plants, and alien invasive plants. The alien invasive plants are mainly from the Americas. The total number of spontaneous plant species from different sources or present in different life forms first increased and then decreased with a single peak. In addition, most spontaneous plant species had their main growing season in July and August. The number of spontaneous plant species in their flowering seasons also first increased and then decreased, with a single peak from March to November. In addition, more spontaneous plant species were in their flowering seasons in July than at any other time of the year. The monthly average value for the Shannon–Wiener diversity index of spontaneous plant associations in the roadside habitat was the highest, followed by those of the woodland and lakeside habitats, and that of the coastline was the lowest from April to September. The monthly average values for the Shannon–Wiener diversity index of the associations in the four habitats also first increased and then decreased, with a single peak in July during the same season.

There are many species of alien invasive plants, including spontaneous plants, in the park during their flowering seasons from March to November. Effective measures for the wise use of spontaneous vegetation should be implemented, protecting the diversity of spontaneous plants, and averting the harm caused by alien invasive plant species in the park.

## Figures and Tables

**Table 1:** The associations of spontaneous plants in different habitats of the Tangdao Bay National Wetland Park.

Habitat	Number of Associations	Association
Roadside	37	Ass. *Rumex dentatus*, Ass. *Lagedium sibiricum*, Ass. *Erigeron annuus*, Ass. *Artemisia argyi*, Ass. *Stellaria uliginosa*, Ass. *Cirsium setosum*, Ass. *Artemisia fauriei*, Ass. *Phragmites australis*, Ass. *Descurainia sophia*, Ass*. Sonchus arvensis*, Ass. *Inula japonica*, Ass. *Galinsoga parviflora*, Ass. *Setaria viridis*, Ass. *Erigeron acer*, Ass. *Rumex patientia*, Ass. *Chenopodium album*, Ass. *Xanthium sibiricum*, Ass. *Oxalis pes-caprae*, Ass. *Duchesnea indica*, Ass. *Acalypha australis*, Ass. *Digitaria sanguinalis*, Ass. *Leonurus artemisia*, Ass. *Gaura parviflora*, Ass. *Vigna minima*, Ass. *Coreopsis lanceolata*, Ass. *Pharbitis purpurea*, Ass. *Helianthus tuberosus*, Ass. *Cassia nomame*, Ass. *Imperata cylindrica*, Ass. *Suaeda salsa*, Ass. *Artemisia annua*, Ass. *Oenothera biennis*, Ass. *Eleusine indica*, Ass. *Bidens pilosa*, Ass. *Triarrhena sacchariflora*, Ass. *Viola prionantha*, Ass. *Aster subulatus*
Woodland	25	Ass. *Imperata cylindrica*, Ass. *Artemisia capillaris*, Ass. *Veronica persica*, Ass. *Equisetum arvense*, Ass. *Bromus japonicus*, Ass. *Artemisia sylvatica*, Ass. *Rumex dentatus*, Ass. *Artemisia argyi*, Ass. *Ixeridium chinense*, Ass. *Setaria viridis*, Ass. *Commelina communis*, Ass. *Erigeron annuus*, Ass. *Geranium carolinianum*, Ass. *Erigeron acer*, Ass. *Inula japonica*, Ass. *Cirsium japonicum*, Ass. *Acalypha australis*, Ass. *Leonurus artemisia*, Ass. *Arthraxon hispidus*, Ass. *Kummerowia striata*, Ass. *Trifolium repens*, Ass. *Phytolacca acinosa*, Ass. *Bidens pilosa*, Ass. *Eragrostis pilosa*, Ass. *Miscanthus sinensis*
Lakeside	27	Ass. *Euphorbia helioscopia*, Ass. *Lagedium sibiricum*, Ass. *Setaria viridis*, Ass. *Vigna minima*, Ass. *Helianthus tuberosus*, Ass. *Bidens pilosa*, Ass. *Phragmites australis*, Ass. *Bromus japonicus*, Ass. *Artemisia argyi*, Ass. *Trifolium repens*, Ass. *Erigeron annuus*, Ass. *Cirsium japonicum*, Ass. *Ixeris polycephala*, Ass. *Bidens biternata*, Ass. *Oenothera biennis*, Ass. *Digitaria sanguinalis*, Ass. *Imperata cylindrica*, Ass*. Inula japonica*, Ass. *Commelina communis*, Ass. *Artemisia capillaris*, Ass. *Melilotus officinalis*, Ass. *Chenopodium glaucum*, Ass. *Echinochloa phyllopogon*, Ass. *Themeda japonica*, Ass. *Erigeron acer*, Ass. *Chenopodium album*, Ass. *Triarrhena sacchariflora*
Coastline	18	Ass. *Artemisia argyi*, Ass. *Imperata cylindrica*, Ass. *Swertia veratroides*, Ass. *Erigeron annuus*, Ass. *Sonchus oleraceus*, Ass. *Phragmites australis*, Ass. *Bromus japonicus*, Ass. *Suaeda salsa*, Ass. *Polygonum sibiricum*, Ass. *Calystegia soldanella*, Ass. *Sonchus arvensis*, Ass. *Rumex patientia*, Ass. *Cynodon dactylon*, Ass. *Aeluropus sinensis*, Ass. *Setaria viridis*, Ass. *Salsola collina*, Ass. *Cirsium japonicum*, Ass. *Triarrhena sacchariflora*

**Table 2:** The family and numbers of genera and species in different habitats.

Family	Roadside	Lakeside	Woodland	Coastline	Family	Roadside	Lakeside	Woodland	Coastline
Asteraceae	22/37	10/17	10/17	5/5	Oxalidaceae	1/1	1/1	1/1	—
Gramineae	18/21	12/13	10/11	2/2	Rubiaceae	1/1	1/1	2/2	—
Leguminosae	8/10	4/4	2/4	—	Commelinaceae	1/1	1/1	1/1	—
Caryophyllaceae	6/9	3/3	4/5	1/1	Crassulaceae	1/1	1/1	—	—
Brassicaceae	6/9	2/2	4/4	—	Plumbaginaceae	1/1	—	—	1/1
Polygonaceae	2/7	2/4	2/3	2/7	Equisetaceae	—	1/1	1/2	—
Rosaceae	2/4	2/4	3/3	1/1	Liliaceae	1/1	—	—	—
Cyperaceae	3/4	3/4	1/1	—	Celastraceae	1/1	—	—	—
Convolvulaceae	3/4	—	1/2	—	Ulmaceae	1/1	—	—	—
Chenopodiaeae	1/3	1/2	1/2	3/3	Tamaricaceae	1/1	—	—	—
Plantaginaceae	1/3	1/2	1/2	3/3	Portulacaceae	1/1	—	—	—
Scrophulariaceae	3/3	1/1	2/2	1/1	Apocynaceae	—	1/1	—	—
Boraginaceae	3/3	1/1	1/1	1/1	Typhaceae	—	1/1	—	—
Asclepiadaceae	2/3	2/3	2/2	—	Alismataceae	—	1/1	—	—
Geraniaceae	2/3	2/3	1/1	—	Potamogetonaceae	—	1/1	—	—
Euphorbiaceae	1/3	2/3	1/2	—	Phytolaccaceae	—	—	1/1	—
Moraceae	2/2	2/2	1/1	—	Malvaceae	—	—	1/1	—
Amaranthaceae	2/3	—	1/1	—	Cruciferae	—	—	1/1	—
Onagraceae	2/2	1/1	1/1	—	Vitaceae	—	—	1/1	—
Primulaceae	2/2	—	1/1	—	Aristolochiaceae	—	—	1/1	—
Solanaceae	2/2	—	1/1	—	Aizoaceae	—	—	—	1/1
Labiatae	1/1	2/2	1/1	1/1	Total	105/149	63/81	63/80	22/27
Violaceae	1/1	1/1	1/1	—					

**Table 3:** Invasion level, invasion source, and life forms of alien invasive plants in the exhibition and education area of the Tangdao Bay National Wetland Park.

Family	Genera	Species	Invasion Level	Invasion Source	Live Form
I Asteraceae	1 *Erigeron*	(1) *Erigeron annuus*	1	The Americas	Annual or biennial herb
	2 *Sonchus*	(2) *Sonchus oleraceus*	4	Europe and central Asia	Annual or biennial herb
		(3) *S. asper*	4	Europe	Annual herb
	3 *Coreopsis*	(4) *Coreopsis lanceolata*	3	North America	Perennial herb
	4 *Conyza*	(5) *Conyza bonariensis*	2	South America	Annual or biennial herb
	5 *Helianthus*	(6) *Helianthus tuberosus*	4	North America	Annual herb
	6 *Galinsoga*	(7) *Galinsoga parviflora*	2	South America	Annual herb
	7 *Bidens*	(8) *Bidens pilosa*	1	Americas	Annual herb
		(9) *B. bipinnata*	3	Americas	Annual herb
	8 *Aster*	(10) *Aster subulatus*	1	North America	Annual herb
	9 *Eclipta*	(11) *Eclipta prostrata*	4	The Americas	Annual herb
	10 *Solidago*	(12) *Solidago canadensis*	1	North America	Perennial herb
II Gramineae	11 *Lolium*	(13) *Lolium perenne*	4	Europe	Annual herb
	12 *Chloris*	(14) *Chloris virgata*	4	Africa	Annual herb
III Leguminosae	13 *Medicago*	(15) *Medicago sativa*	4	West Asia	Perennial herb
	14 *Amorpha*	(16) *Amorpha fruticosa*	5	North America	Woody plant (shrub)
	15 *Trifolium*	(17) *Trifolium repens*	2	Europe	Perennial herb
	16 *Melilotus*	(18) *Melilotus officinalis*	4	West Asia	Perennial herb
IV Brassicaceae	17 *Coronopus*	(19) *Coronopus didymus*	4	South America	Annual or biennial herb
	18 *Lepidium*	(20) *Lepidium virginicum*	2	North America	Annual or biennial herb
	19 *Capsella*	(21) *Capsella bursa-pastoris*	4	West Asia, Europe	Annual or biennial herb
V Caryophyllaceae	20 *Myosoton*	(22) *Myosoton aquaticum*	4	Europe	Perennial herb
	21 *Ceratium*	(23) *Ceratium glomeratum*	3	Europe	Annual herb
VI Cyperaceae	22 *Cyperus*	(24) *Cyperus rotundus*	4	India	Perennial herb
VII Chenopodiaeae	23 *Chenopodium*	(25) *Chenopodium serotinum*	4	Europe	Annual herb
		(26) *C. glaucum*	4	uncertain	Annual herb
VIII Convolvulaceae	24 *Pharbitis*	(27) *Pharbitis purpurea*	1	The Americas	Annual herb
IX Euphorbiaceae	25 *Euphorbia*	(28) *Euphorbia maculata*	3	North America	Annual herb
	26 *Ricinus*	(29) *Ricinus communis*	2	Africa	Annual herb
X Solanaceae	27 *Datura*	(30) *Datura stramonium*	2	North America	Annual herb
XI Amaranthaceae	28 *Amaranthus*	(31) *Amaranthus retroflexus*	1	The Americas	Annual herb
		(32) *A. viridis*	2	South America	Annual herb
	29 *Celosia*	(33) *Celosia argentea*	2	India	Annual herb
XII Malvaceae	30 *Abutilon*	(34) *Abutilon theophrasti*	3	India	Annual herb
XIII Onagraceae	31 *Oenothera*	(35) *Oenothera biennis*	2	North America	Perennial herb
	32 *Gaura*	(36) *Gaura parviflora*	2	North America	Annual herb
XIV Scrophulariaceae	33 *Veronica*	(37) *Veronica persica*	3	West Asia	Annual or biennial herb
XV Plantaginaceae	34 *Plantago*	(38) *Plantago lanceolata*	3	Europe	Annual herb
XVI Geraniaceae	35 *Geranium*	(39) *Geranium carolinianum*	2	North America	Annual herb

**Table 4:** Number of species of spontaneous plants in different habitats of the Tangdao Bay National Wetland Park.

Season	Roadside	Woodland	Lakeside	Coastline
Growing season	150	80	82	24
September	127	76	75	21

**Table 5:** Comparison of the number of alien invasive plant species in the Tangdao Bay National Wetland Park with those of other wetland examples in China.

Wetland	Area (hm^2^)	Family/Genera/ Species
Tangdao Bay National Wetland Park	1638	16/35/39
Dahuangpu Provincial Nature Reserve of Tianjin City	10,465	13/21/27
Wetland of Sishui County, Ji’ning City, Shandong Province	—	14/26/39
Wetland of Luoyang City, Henan Province	—	18/35/49
Xin’anjiang Reservoir Wetland of Hangzhou City	58,000	22/43/55
Hangzhou Xixi Natioanl Wetland Park	1008	27/52/69
West Lake Wetland of Hangzhou City	636	20/27/30
Qiantangjiang Estuary Wetland of Hangzhou City	20,033	20/41/51
Fuchunjiang Reservoir Wetland of Hangzhou City	4336	20/32/36
Hangzhou section of Beijing–Hangzhou Canal Wetland	589	15/20/24
Qianmutian Marsh Welland of Hangzhou City	38	8/13/15

## Data Availability

Not applicable.

## Supplementary Materials

The following supporting information can be downloaded at: https://www.mdpi.com/article/10.3390/ijerph191811665/s1. Table S1. The spontaneous plants and their sources, life form in Tangdao Bay National Wetland Park, Qingdao City of China.
