# Supplementary material for: Biodiversity and Spatiotemporal Distribution of Spontaneous Vegetation in Tangdao Bay National Wetland Park, Qingdao City, China
Source: Int J Environ Res Public Health. 2022 Sep 16;19(18):11665. doi: 10.3390/ijerph191811665 (PMC9517414; doi:10.3390/ijerph191811665)
Supplement: Supplementary file 1 [file ijerph-19-11665-s001.zip › ijerph-1866306-supplementary.pdf]

Supplementary Table S1. The spontaneous plants and their sources, life form in Tangdao Bay National Wetland Park, Qingdao City of China.

Family	Genus	Species	Source of species	Life form
I Asteraceae	1. *Erigeron*	(1) *Erigeron annuus*	alien invasive plant	annual or biennial herb
		(2) *Erigeron acer*	native plant	biennial herb
	2. *Hemistepta*	(3) *Hemistepta lyrata*	native plant	annual herb
	3. *Ixeridium*	(4) *Ixeridium sonchifolia*	alien introduced plant	perennial herb
		(5) *Ixeridium dentatum*	native plant	perennial herb
		(6) *Ixeridium chinense*	native plant	perennial herb
		(7) *Ixeridium gramineum*	native plant	perennial herb
		(8) *Ixeridium biparum*	native plant	annual herb
	4. *Ixeris*	(9) *Ixeris polycephala*	domestic introduced plant	annual herb
	5. *Artemisia*	(10) *Artemisia mongolica*	native plant	perennial herb
		(11) *Artemisia capillaries*	native plant	annual herb
		(12) *Artemisia annua*	native plant	annual herb
		(13) *Artemisia argyi*	native plant	perennial herb
		(14) *Artemisia fauriei*	native plant	perennial herb
		(15) *Artemisia princeps*	native plant	perennial herb
		(16) *Artemisia sylvatica*	native plant	perennial herb
		(17) *Artemisia carvifolia*	native plant	annual herb
	6. *Youngia*	(18) *Youngia Japonica*	native plant	annual or biennial herb
	7. *Taraxacum*	(19) *Taraxacum mongolicum*	native plant	perennial herb
	8. *Sonchus*	(20) *Sonchus oleraceus*	alien invasive plant	annual or biennial herb
		(21) *Sonchus brachyotus*	native plant	annual herb
		(22) *Sonchus arvensis*	native plant	perennial herb
		(23) *Sonchus asper*	alien invasive plant	annual herb
	9. *Cirsium*	(24) *Cirsium setosum*	native plant	perennial herb

		(25) *Cirsium japonicum*	native plant	annual herb
	10. *Kalimeris*	(26) *Kalimeris indica*	native plant	perennial herb
	11. *Coreopsis*	(27) *Coreopsis lanceolata*	alien invasive plant	perennial herb
	12. *Conyza*	(28) *Conyza bonariensis*	alien invasive plant	annual or biennial herb
	13. *Lagedium*	(29) *Lagedium sibiricum*	domestic introduced plant	annual or biennial herb
	14. *Scorzonera*	(30) *Scorzonera albicaulis*	native plant	perennial herb
	15. *Helianthus*	(31) *Helianthus tuberosus*	alien invasive plant	annual herb
	16. *Galinsoga*	(32) *Galinsoga parviflora*	alien invasive plant	annual herb
	17. *Bidens*	(33) *Bidens pilosa*	alien invasive plant	annual herb
		(34) *Bidens parviflora*	native plant	annual herb
		(35) *Bidens biternata*	native plant	annual herb
		(36) *Bidens bipinnata*	alien invasive plant	annual herb
	18. *Inula*	(37) *Inula japonica*	native plant	perennial herb
	19. *Xanthium*	(38) *Xanthium sibiricum*	native plant	annual herb
	20. *Aster*	(39) *Aster subulatus*	alien invasive plant	annual herb
	21. *Eclipta*	(40) *Eclipta prostrata*	alien invasive plant	annual herb
	22. *Solidago*	(41) *Solidago canadensis*	alien invasive plant	perennial herb
	23. *Heteropappu*	(42) *Heteropappus hispidus*	native plant	annual or biennial herb
	24. *Paraixeris*	(43) *Paraixeris pinnatipartita*	native plant	annual herb
II Brassicaceae	25. *Orychophragmus*	(44) *Orychophragmus violaceus*	native plant	annual or biennial herb
	26. *Capsella*	(45) *Capsella bursa-pastoris*	alien invasive plant	annual or biennial herb
	27. *Descurainia*	(46) *Descurainia sophia*	native plant	annual herb
	28. *Cardamine*	(47) *Cardamine impatien*	native plant	annual or biennial herb
		(48) *Cardamine hirsuta*	native plant	annual herb
	29. *Coronopus*	(49) *Coronopus didymus*	alien invasive plant	annual or biennial herb
	30. *Lepidium*	(50) *Lepidium virginicum*	alien invasive plant	annual or biennial herb

		(51) *Lepidium apetalum*	native plant	annual or biennial herb
	31. *Rorippa*	(52) *Rorippa globosa*	native plant	annual or biennial herb
		(53) *Rorippa islandica*	native plant	annual or biennial herb
		(54) *Rorippa indica*	native plant	annual or biennial herb
	32. *Camelina*	(55) *Camelina microcarpa*	native plant	annual herb
	33. *Erysimum*	(56) *Erysimum cheiranthoides*	native plant	annual or biennial herb
III Gramineae	34. *Eleusine*	(57) *Eleusine indica*	domestic introduced plant	annual herb
	35. *Poa*	(58) *Poa pratensis*	native plant	perennial herb
		(59) *Poa annua*	native plant	annual herb
	36. *Bromus*	(60) *Bromus japonicas*	native plant	perennial herb
	37. *Phragmites*	(61) *Phragmites australis*	native plant	perennial herb
	38. *Imperata*	(62) *Imperata cylindrica*	native plant	perennial herb
	39. *Cynodon*	(63) *Cynodon dactylon*	domestic introduced plant	perennial herb
	40. *Digitaria*	(64) *Digitaria sanguinalis*	native plant	annual herb
		(65) *Digitaria ischaemum*	native plant	annual herb
	41. *Avena*	(66) *Avena sativa*	native plant	annual herb
	42. *Spodiopogon*	(67) *Spodiopogon sibiricus*	native plant	perennial herb
	43. *Themeda*	(68) *Themeda japonica*	native plant	perennial herb
	44. *Zoysia*	(69) *Zoysia japonica*	native plant	perennial herb
	45. *Achnatherum*	(70) *Achnatherum extremiorientale*	native plant	perennial herb
	46. *Polypogon*	(71) *Polypogon fugax*	native plant	annual herb
	47. *Setaria*	(72) *Setaria viridis*	native plant	annual herb
		(73) *Setaria glauca*	native plant	annual herb
	48. *Aeluropus*	(74) *Aeluropus sinensis*	native plant	perennial herb
	49. *Alopecurus*	(75) *Alopecurus aequalis*	native plant	annual herb
	50. *Lolium*	(76) *Lolium perenne*	alien invasive plant	annual herb

	51. *Arthraxon*	(77) *Arthraxon hispidus*	native plant	annual herb
	52. *Chloris*	(78) *Chloris virgata*	alien invasive plant	annual herb
	53. *Triarrhena*	(79) *Triarrhena sacchariflora*	native plant	perennial herb
	54. *Cymbopogon*	(80) *Cymbopogon goeringii*	native plant	perennial herb
	55. *Eragrostis*	(81) *Eragrostis pilosa*	native plant	annual herb
	56. *Calamagrostis*	(82) *Calamagrostis epigeios*	native plant	perennial herb
	57. *Miscanthus*	(83) *Miscanthus sinensis*	native plant	perennial herb
	58. *Leptochloa*	(84) *Leptochloa chinensis*	native plant	annual herb
	59. *Sporobolus*	(85) *Sporobolus fertilis*	native plant	perennial herb
	60. *Bothriochloa*	(86) *Bothriochloa ischaemum*	native plant	perennial herb
	61. *Cleistogenes*	(87) *Cleistogenes caespitosa*	native plant	perennial herb
	62. *Echinochloa*	(88) *Echinochloa phyllopogon*	domestic introduced plant	annual herb
IV Caryophyllaceae	63. *Cleistogenes*	(89) *Stellaria uliginosa*	domestic introduced plant	biennial herb
		(90) *Stellaria chinensis*	native plant	perennial herb
		(91) *Stellaria media*	native plant	annual or biennial herb
	64. *Ceratium*	(92) *Ceratium glomeratum*	alien invasive plant	annual herb
	65. *Arenaria*	(93) *Arenaria serpyllifolia*	native plant	annual or biennial herb
	66. *Silene*	(94) *Silene aprica*	native plant	annual or biennial herb
	67. *Gypsophila*	(95) *Gypsophila oldhamiana*	native plant	perennial herb
	68. *Myosoton*	(96) *Myosoton aquaticum*	alien invasive plant	perennial herb
V Scrophulariaceae	69. *Veronica*	(97) *Veronica persica*	alien invasive plant	annual or biennial herb
	70. *Mazus*	(98) *Mazus japonicas*	native plant	annual herb
	71. *Paulowinia*	(99) *Paulowinia fortunei*	domestic introduced plant	woody plant/tree
VI Rosaceae	72. *Potentilla*	(100) *Potentilla freyniana*	native plant	perennial herb
		(101) *Potentilla supina*	native plant	annual or biennial herb
		(102) *Potentilla discolor*	native plant	perennial herb

	73. *Sanguisorba*	(103) *Sanguisorba officinalis*	native plant	perennial herb
	74. *Armeniaca*	(104) *Armeniaca vulgaris*	native plant	woody plant/tree
	75. *Rubus*	(105) *Rubus parvifolius*	native plant	annual herb
	76. *Duchesnea*	(106) *Duchesnea indica*	native plant	perennial herb
	77. *Rosa*	(107) *Rosa multiflora*	native plant	woody plant/shrub
VII Boraginaceae	78. *Bothriospermum*	(108) *Bothriospermum chinense*	native plant	annual herb
	79. *Messerschmidia*	(109) *Messerschmidia sibirica*	native plant	perennial herb
	80. *Trigonotis*	(110) *Trigonotis peduncularis*	native plant	annual herb
VIII Plantaginaceae	81. *Plantago*	(111) *Plantago depressa*	native plant	annual or biennial herb
		(112) *Plantago lanceolata*	alien invasive plant	annual herb
		(113) *Plantago asiatica*	native plant	perennial herb
IX Polygonaceae	82. *Rumex*	(114) *Rumex dentatus*	native plant	annual herb
		(115) *Rumex acetosa*	native plant	perennial herb
		(116) *Rumex patientia*	native plant	perennial herb
		(117) *Polygonum perfoliatum*	native plant	annual herb
	83. *Polygonum*	(118) *Polygonum sibiricum*	native plant	perennial herb
		(119) *Polygonum aviculare*	native plant	annual herb
		(120) *Polygonum lapathifolium*	native plant	annual herb
		(121) *Polygonum plebeium*	native plant	annual herb
		(122) *Polygonum hydropipe*	native plant	annual herb
X Leguminosae	84. *Medicago*	(123) *Medicago sativa*	alien invasive plant	perennial herb
		(124) *Medicago lupulina*	native plant	perennial herb
	85. *Vicia*	(125) *Vicia bungei Ohwi*	native plant	annual herb
	86. *Gueldenstaedtia*	(126) *Gueldenstaedtia maritima*	native plant	perennial herb
		(127) *Gueldenstaedtia verna*	native plant	perennial herb
	87. *Lespedeza*	(128) *Amorpha fruticosa*	alien invasive plant	woody plant/shrub

		(129) *Lespedeza bicolor*	native plant	woody plant/shrub
	88. *Trifolium*	(130) *Trifolium repens*	alien invasive plant	perennial herb
	89. *Vicia*	(131) *Vicia angustifolia*	native plant	annual or biennial herb
		(132) *Vicia amoena*	native plant	perennial herb
	90. *Pueraria*	(133) *Pueraria lobata*	native plant	perennial herb
	91. *Melilotus*	(134) *Melilotus officinalis*	alien invasive plant	perennial herb
	92. *Vigna*	(135) *Vigna minima*	native plant	annual herb
	93. *Kummerowia*	(136) *Kummerowia striata*	native plant	annual herb
	94. *Cassia*	(137) *Cassia nomame*	native plant	annual herb
XI Violaceae	95. *Viola*	(138) *Viola prionantha*	native plant	perennial herb
		(139) *Viola philippica*	native plant	perennial herb
XII Oxalidaceae	96. *Oxalis*	(140) *Oxalis pes-caprae*	alien introduced plant	perennial herb
XIII Rubiaceae	97. *Rubia*	(141) *Rubia cordifolia*	native plant	perennial herb
	98. *Galium*	(142) *Galium aparine*	native plant	perennial herb
	99. *Paederia*	(143) *Paederia scandens*	native plant	perennial herb
XIV Euphorbiaceae	100. *Euphorbia*	(144) *Euphorbia helioscopia*	domestic introduced plant	annual or biennial herb
		(145) *Euphorbia esula*	native plant	perennial herb
		(146) *Euphorbia maculata*	alien invasive plant	annual herb
	101. *Acalypha*	(147) *Acalypha australis*	native plant	annual herb
	102. *Ricinus*	(148) *Ricinus communis*	alien invasive plant	annual herb
XV Geraniaceae	103. *Erodium*	(149) *Erodium stephanianum*	native plant	perennial herb
	104. *Geranium*	(150) *Geranium carolinianum*	alien invasive plant	annual herb
		(151) *Geranium wlassowianum*	native plant	perennial herb
XVI Labiatae	105. *Lamium*	(152) *Lamium amplexicaule*	domestic introduced plant	annual or biennial herb
	106. *Leonurus*	(153) *Leonurus artemisia*	native plant	annual or biennial herb
	107. *Scutellaria*	(154) *Scutellaria strigillosa*	native plant	perennial herb

	108. *Salvia*	(155) *Salvia plebeia*	native plant	annual or biennial herb
XVII Asclepiadaceae	109. *Cynanchum*	(156) *Cynanchum chinense*	native plant	perennial herb
		(157) *Cynanchum thesiodes*	native plant	perennial herb
	110. *Metaplexis*	(158) *Metaplexis japonica*	native plant	perennial herb
XVIII Chenopodiaceae	111. *Chenopodium*	(159) *Chenopodium album*	native plant	annual herb
		(160) *Chenopodium serotinum*	alien invasive plant	annual herb
		(161) *Chenopodium glaucum*	alien invasive plant	annual herb
		(162) *Chenopodium gracilispicum*	native plant	annual herb
	112. *Suaeda*	(163) *Suaeda salsa*	native plant	annual herb
	113. *Salsola*	(164) *Salsola collina*	native plant	annual herb
XIX Cyperaceae	114. *Cyperus*	(165) *Cyperus rotundus*	alien invasive plant	perennial herb
		(166) *Cyperus amuricus*	native plant	annual herb
	115. *Carex*	(167) *Carex breviculmis*	native plant	perennial herb
		(168) *Scirpus validus*	native plant	perennial herb
	116. *Scirpus*	(169) *Scirpus yagara*	native plant	perennial herb
		(170) *Carex kobomugi*	native plant	perennial herb
	117. *Pycreus*	(171) *Pycreus globosus*	native plant	annual herb
XX Crassulaceae	118. *Rhodiola*	(172) *Floret Rhodiola*	native plant	perennial herb
XXI Primulaceae	119. *Lysimachia*	(173) *Lysimachia pentapetala*	native plant	annual herb
	120. *Androsace*	(174) *Androsace incana*	native plant	annual or biennial herb
XXII Celastraceae	121. *Euonymus*	(175) *Euonymus fortunei*	native plant	perennial herb
XXIII Moraceae	122. *Humulus*	(176) *Humulus scandens*	native plant	perennial herb
	123. *Broussonetia*	(177) *Broussonetia papyrifera*	native plant	woody plant/tree
XXIV Convolvulaceae	124. *Calystegia*	(178) *Calystegia pellita*	native plant	perennial herb
		(179) *Calystegia sepium*	native plant	perennial herb
		(180) *Calystegia soldanella*	native plant	perennial herb

	125. *Pharbitis*	(181) *Pharbitis purpurea*	alien invasive plant	annual herb
	126. *Convolvulus*	(182) *Convolvulus arvensis*	native plant	perennial herb
XXV Apocyanceae	127. *Apocynum*	(183) *Apocynum venetum*	native plant	perennial herb
XXVI Phytolaccaceae	128. *Phytolacca*	(184) *Phytolacca acinosa*	native plant	perennial herb
XXVII Equisetaceae	129. *Equisetum*	(185) *Equisetum arvense*	native plant	perennial herb
		(186) *Equisetum ramosissimum*	native plant	perennial herb
XXVIII Ulmaceae	130. *Ulmus*	(187) *Ulmus pumila*	native plant	woody plant/tree
XXIX Typhaceae	131. *Typha*	(188) *Typha orientalis*	native plant	perennial herb
		(189) *Typha angustata*	native plant	perennial herb
XXX Tamaricaceae	132. *Tamarix*	(190) *Tamarix chinensis*	native plant	woody plant/tree or shrub
XXXI Commelinaceae	133. *Commelina*	(191) *Commelina communis*	native plant	annual herb
XXXII Plumbaginaceae	134. *Limonium*	(192) *Limonium bicolor*	native plant	perennial herb
XXXIII Solanaceae	135. *Lycium*	(193) *Lycium chinense*	native plant	woody plant/shrub
	136. *Datura*	(194) *Datura stramonium*	alien invasive plant	annual herb
	137. *Solanum*	(195) *Solanum cathayanum*	native plant	perennial herb
XXXIV Alismataceae	138. *Alisma*	(196) *Alisma plantago-aquatica*	native plant	perennial herb
XXXV Portulacaceae	139. *Portulaca*	(197) *Portulaca oleracea*	native plant	annual herb
XXXVI Amaranthaceae	140. *Amaranthus*	(198) *Amaranthus retroflexus*	alien invasive plant	annual herb
		(199) *Amaranthus viridis*	alien invasive plant	annual herb
	141. *Celosia*	(200) *Celosia argentea*	alien invasive plant	annual herb
	142. *Achyranthes*	(201) *Achyranthes bidentata*	native plant	perennial herb
XXXVII Verbenaceae	143. *Vitex*	(202) *Vitex negundo*	native plant	woody plant/tree or shrub
XXXVIII Malvaceae	144. *Abutilon*	(203) *Abutilon theophrasti*	alien invasive plant	annual herb
XXXIX Aristolochiaceae	145. *Aristolochia*	(204) *Aristolochia debilis*	native plant	perennial herb
XL Potamogetonaceae	146. *Potamogeton*	(205) *Potamogeton crispus*	native plant	perennial herb
XLI Aizoaceae	147. *Mollugo*	(206) *Mollugo stricta*	native plant	annual herb

XLII Onagraceae	148. *Oenothera*	(207) *Oenothera biennis*	alien invasive plant	perennial herb
	149. *Gaura*	(208) *Gaura parviflora*	alien invasive plant	annual herb
XLIII Liliaceae	150. *Allium*	(209) *Allium macrostemon*	native plant	perennial herb
XLIV Vitaceae	151. *Cayratia*	(210) *Cayratia japonica*	native plant	perennial herb
